# Semi-arid grasses combine contrasting strategies of dehydration tolerance associated with carbohydrate storage and embolism resistance under drought

**DOI:** 10.1093/jxb/erag003

**Published:** 2026-02-24

**Authors:** Maria Dolores Hidalgo-Galvez, Chaïa Akhoun-Piernicka, Gerónimo A Cardozo, Annette Morvan-Bertrand, Marie-Pascale Prud’homme, Karim Barkaoui, Florence Volaire

**Affiliations:** Universidad de Sevilla, Seville E-41012, Spain; CEFE, CNRS, INRAE, EPHE, IRD, Université de Montpellier, Montpellier, France; Mediterranean Center for Environmental Studies (CEAM Foundation), Joint Research Unit University of Alicante-CEAM, University of Alicante, San Vicente del Raspeig, Alicante E-03690, Spain; CEFE, CNRS, INRAE, EPHE, IRD, Université de Montpellier, Montpellier, France; CEFE, CNRS, INRAE, EPHE, IRD, Université de Montpellier, Montpellier, France; National Institute of Agriculture Research (INIA), Pasture and Forage Area, Treinta y Tres, Uruguay; UNICAEN, INRAE, EVA, University of Normandy, Caen F-14000, France; UNICAEN, INRAE, EVA, University of Normandy, Caen F-14000, France; CIRAD, UMR AMAP, Montpellier F-34398, France; AMAP, CIRAD, CNRS, INRAE, IRD, Université de Montpellier, Montpellier, France; CEFE, CNRS, INRAE, EPHE, IRD, Université de Montpellier, Montpellier, France; INRAE-Montpellier, France

**Keywords:** *Dactylis* subspecies, drought survival, fructans, leaf meristems, P_50_, *Stipa* species, water-soluble carbohydrates, xylem vulnerability

## Abstract

Dehydration tolerance and embolism resistance contribute to plant drought survival, but their limits and combinations in perennial grasses remain unexplored. We investigated four grasses (*Stipa* species and *Dactylis* subspecies) from Mediterranean sites prone to intense summer drought. Plant tolerance to soil water deficit and tissue dehydration, embolism resistance (P_50_), and water-soluble carbohydrates (WSCs) were measured under severe drought. *Dactylis* were more dehydration tolerant, reaching 50% survival at a lower soil water potential (−6.07 MPa) than *Stipa* (−2.64 MPa), and at a lower leaf base water content (32.8%) than *Stipa* (50.0%). *Dactylis* accumulated higher WSCs in leaf bases [479 mg g^−1^ dry mass (DM), high fructan concentration] than *Stipa* (17 mg g^−1^ DM, high sucrose concentration). WSCs contributed 47% (*Dactylis*) and 29% (*Stipa*) to osmotic adjustment. However, *Stipa* had higher embolism resistance (P_50_= −8.7 MPa) than *Dactylis* (−2.9 MPa). Plants from the most arid sites had the highest dehydration tolerance, while embolism resistance was uncorrelated with aridity of the sites of origin. We found the highest embolism resistance and dehydration tolerance reported for herbaceous species. We showed that semi-arid grasses combine contrasting strategies to survive drought. Assessing these strategy combinations is crucial to predicting plant resilience under climate change.

## Teaser

To survive drought, semi-arid grasses *Dactylis* and *Stipa* exhibit extreme strategies, combined differently; dehydration tolerance associated with fructan accumulation was shown to be uncorrelated to embolism resistance.

## Introduction

Climate change increases temperatures and alters precipitation patterns, enhancing the frequency of drought and extreme events (IPCC, 2021). In Mediterranean areas, longer and more intense dry summers are expected (Tramblay *et al*., 2020). In these regions, grasslands and rangelands account for nearly half of the vegetation cover of 270 Mha (Hervieu and Thibault, 2009). They provide numerous ecosystem services, such as the maintenance of biodiversity, pasture production for livestock, and soil protection against erosion, and play a role in the global carbon and water cycles (Zhao *et al*., 2020). Climate change is one of the most critical threats to the survival of perennial grass species (Poirier *et al*., 2012; Ehrlén, 2019), resulting in long-term degradation of these ecosystems (Hovenden *et al*., 2017; Winkler *et al*., 2019). Therefore, understanding plant drought-induced mortality has become crucial for anticipating the response of these ecosystems to climate change and evaluating their long-term resilience to extreme events (Mitchell *et al*., 2016; Norton *et al*., 2016).

To survive severe droughts, plants combine various strategies, including dehydration tolerance and embolism resistance (Volaire, 2018). Dehydration tolerance enables plants to survive at a low level of tissue hydration (Volaire, 2018). In perennial grasses, the term ‘dehydration tolerance’ can refer both to plant responses to soil water deficit and to the capacity of key organs, the leaf meristems, to withstand tissue dehydration since both are highly correlated (Barkaoui and Volaire, 2023). To compare species/populations and/or sites/experiments, dehydration tolerance can be assessed using the standardized lethal drought index (LD_50_), which corresponds to the soil water potential associated with 50% plant mortality. In perennial grasses, the youngest leaf tissues in leaf bases, including meristems, survive water stress better than most mature plant tissues (Munns *et al*., 1979; Barlow *et al*., 1980). Indeed, leaf meristems are a strong sink for water and carbohydrates throughout severe stress (Spollen and Nelson, 1994). The accumulation of solutes such as water-soluble carbohydrates (WSCs), particularly fructans (soluble sucrose-derived fructose polymers), contributes to dehydration tolerance by osmotic adjustment, and also cell membrane protection during tissue dehydration, thus ensuring plant survival through the protection of leaf meristems (Hincha *et al*., 2007; Volaire *et al*., 2014, 2020; Zwicke *et al*., 2015).

Under severe drought, plant water flow is interrupted by the embolization of the xylem—when gas formation interrupts water transport and potentially affects survival of the plant (Charrier *et al*., 2021; Jacob *et al*., 2022). Hence, embolism resistance, and therefore water transport capacity, is regarded as a key trait for drought survival. Embolism resistance is assessed by the leaf water potential leading to 50% or 88% embolism—P_50_ or P_88_ (Tyree and Zimmermann, 2002; Lens *et al*., 2016; Volaire *et al*., 2018). A large variation in P_50_ was found in woody species and was associated with the aridity of their origin site. The most extreme value has been found (almost −19 MPa) for *Callitris tuberculata*, a species highly adapted to extreme droughts, with xylem pressure approaching the most negative recorded limits for water transport in plants (Larter *et al*., 2017). Interestingly, the range of variation of P_50_ within temperate grasses (−0.7 MPa to −7.5 MPa) is similar to that found in woody angiosperm species from the same biogeographical areas (Lens *et al*., 2016). The most negative P_50_ value for a perennial grass was recorded at approximately −7.5 MPa for *Stipa pennata* originating from the French Mediterranean region (Lens *et al*., 2016). At the intra-specific level, embolism resistance was highly correlated to the varying levels of dehydration tolerance in summer in populations of *Dactylis glomerata* originating from Mediterranean to northern climates (Bristiel *et al*., 2018; Volaire *et al*., 2018). To further investigate the limits of drought survival in herbaceous species, this study explores the limits and combination of strategies in perennial grasses originating from sites amongst the driest of their biogeographical distributions. The LD_50_ integrates the overall ability of plants to withstand severe dehydration at the whole-plant level, while P_50_ reflects the resistance of the hydraulic system to drought-induced failure. Since perennial grasses rely on both the persistence of meristematic tissues and the maintenance of vascular function to survive extreme droughts, LD_50_ and P_50_ are expected to represent positively correlated strategies to survive drought.

This study tested two species of the genus *Stipa* and two subspecies of the species *D. glomerata*, all perennial grasses typical of Mediterranean grasslands. *Stipa tenacissima* L. is an abundant species found in semi-arid areas (e.g. South-Eastern Spain), with high plasticity allowing for quick responses to any rainfall pulse (Pugnaire and Haase, 1996; Nedjimi, 2018). This species is adapted to severe water stress due to its elongated, narrow, and rolled leaves, and a dense and shallow root system (Bochet *et al*., 1999; El-Abbassi *et al*., 2020; Illuminati *et al*., 2022). In contrast, *Stipa pennata* L. is abundant in the northern Mediterranean basin (e.g. in the Larzac massif, Southern France), where summer drought is usually severe, but winters are mild (Cardozo *et al*., 2024). This xerothermic species has leaves of similar width, but lower tissue density and toughness, and a thinner root system than *S. tenacissima* (Gonzalo *et al*., 2013). *Dactylis glomerata* L. has a wide geographical distribution in Europe and Northern Africa. Mediterranean populations have a higher dehydration tolerance than those from temperate and northern environments (Bristiel *et al*., 2018, 2019; Shihan *et al*., 2022; Barkaoui and Volaire, 2023). Here, we tested *D. glomerata* L. subsp. *hispanica* (Roth) Nyman from Southern Spain (hereafter *D. glomerata hisp.*) and *D. glomerata* from Morocco (hereafter *D. glomerata* Kasbah). These subspecies have thin, broad, and long leaves, as well as numerous thin roots (Beddows, 1959).

This study compared these four perennial grasses in controlled conditions. Plants were grown in small pots to restrict root system expansion and thus avoid the expression of the dehydration avoidance strategy, under controlled conditions (Bristiel *et al*., 2019). Moreover, the study was carried out in spring to prevent the expression of summer dormancy, which is a strategy allowing Mediterranean perennial grasses to survive under chronic severe summer drought (Laude, 1953; Volaire and Norton, 2006). Indeed, *D. glomerata* Kasbah is summer-dormant (Shihan *et al*., 2022). However, we have no data on the level of summer dormancy of *Stipa* species. Therefore, we compared these grasses at a similar vegetative phenological stage, in early spring, when dormancy has not yet been induced, allowing an unbiased assessment of dehydration tolerance. We hypothesized that (i) a higher dehydration tolerance could be associated with a greater accumulation of WSCs, notably fructans, in leaf bases of these grasses; and (ii) a higher dehydration tolerance could be associated with higher embolism resistance. Overall, this study aimed to assess the contribution of the main strategies of perennial grasses from semi-arid areas to survive extreme drought.

## Materials and methods

### Plant material

The study compared four perennial grasses from sites with a range of aridity indexes (AIs) calculated as the ratio MAP/(MAT+10), where MAP is the mean annual precipitation and MAT is the mean annual temperature (De Martonne, 1926): *S. tenacissima* from Alicante (Southeastern Spain, AI=8.9), *S. pennata* from Larzac (Southern France, AI=47), *D. glomerata* Kasbah from Oum-Er-Rbia (Western Morocco, AI=12.4), and *D. glomerata hisp.* from Seville (Southern Spain, AI=17.4) (Supplementary Table S1; Supplementary Fig. S1). Due to the difficulty of germinating their seeds (Dhief *et al*., 2014; Krichen *et al*., 2014, 2017), *Stipa* species were collected in the field by selecting individuals of 20 cm height (on 29 October 2022 for *S. tenacissima* and on 10 November 2022 for *S. pennata*). *Dactylis* subspecies were sown on 13 September 2022 with seeds obtained from the germplasm bank of Lusignan (INRAE).

### Experimental design

This experiment was carried out in a glasshouse at the Centre for Functional and Evolutionary Ecology of Montpellier (CEFE-CNRS), France (43°38'19.76''N; 3°51'50.90''E). The study was carried out in spring to compare plants during the vegetative stage and avoid the expression of summer dormancy; that is, growth reduction or cessation and foliage senescence in summer, irrespective of the soil water content, which depends on the origin of the grasses (Volaire and Norton, 2006). Small-volume pots (4 litres, 16 cm wide, 21.5 cm deep) were used to restrict the expansion of root systems and therefore prevent the expression of a possible dehydration avoidance strategy by using deep soil water reserves. They were filled with the same amount of a standardized substrate composed of 75% sand and 25% soil from the experimental station at CEFE-CNRS. This ensured that all plants had access to exactly the same quantity of soil water reserve at the beginning of the experiment, allowing the temporal dynamics of soil moisture to be accurately measured and compared by weighing the pots (Bristiel *et al*., 2018; Barkaoui and Volaire, 2023).

A set of three plants of each grass, selected to have a similar number of tillers, was transplanted per pot on 7 December 2022 and produced ∼20–30 tillers before the onset of the drought treatment. The 188 pots (45 pots for *S. tenacissima*, 57 pots for *S. pennata*, 42 pots for *D. glomerata* Kasbah, and 44 pots for *D. glomerata hisp.*) were randomly distributed and moved around weekly within the glasshouse to minimize placement effects. Since the growth of potted plants of *S. tenacissima* was not optimal, the experiment was carried out with about half as many pots for this species as for the other grasses. As a result, approximately half as many replicates of the same variables related to dehydration tolerance were obtained for this species. To monitor local climatic variables (Supplementary Fig. S2), a Campbell Datalogger was installed in the glasshouse, recording data every 30 min. The mean temperature was 19.6 °C (range 11–39.5 °C), air relative humidity was 73.4% (range 23–93.9%), and global radiation ranged from 1458.9 µmol m^−2^ s^−1^ (March) to 1602.7 µmol m^−2^ s^−1^ (May). The soil water content (SWC; g H_2_O g^−1^ dry soil) was monitored by gravimetry, with all pots being weighed three times per week (from 30 March 2023 to 30 May 2023; Supplementary Fig. S3). The relationship between SWC (%) and soil water potential (ψs, MPa) of the substrate was calculated following Equation 1:


(1) Ψs=−14.451×exp(−0.487×SWC)

**Fig. 1.**
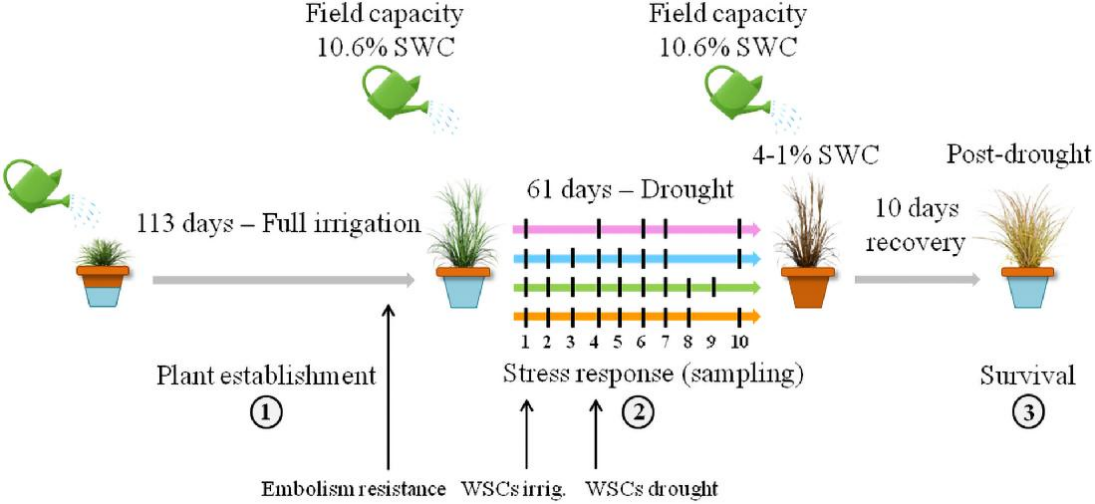
Experimental design for the measurement of dehydration tolerance and embolism resistance in four Mediterranean perennial grasses, *S. tenacissima*, *S. pennata*, *D. glomerata* Kasbah, and *D. glomerata hisp.* (1) ‘Pre-drought stage’. (2) ‘Drought stage’ cessation of irrigation. Sampling (approximately three pots per grass) took place on 10 dates (five for *S. tenacissima* due to fewer pots) as water deficit increased: pink (*S. tenacissima*), blue (*S. pennata*), green (*D. glomerata* Kasbah), and orange (*D. glomerata hisp.*). Measurements of embolism resistance (*n*=4 per grass) and sample collection to analyse water-soluble carbohydrates (WSCs) under irrigation (*n*=5 per grass) and in drought (*n*=5 per grass) are indicated with arrows; (3) ‘Post-drought stage’.

The experiment proceeded through the following three stages (Fig. 1). (i) ‘Pre-drought stage’ (plant establishment): after transplantation, plants were fertilized with 0.15 g of nitrogen every 25 d and once with 150 ml per pot of N-P-K solution (1 ml of Oligodyn:10 litres of water) to enhance establishment. Plants were fully irrigated for 113 d until they were well developed. (ii) ‘Drought stage’ (stress response): on 30 March 2023, pots were stabilized at field capacity (10.6%) so that plants had the same initial amount of available water, and irrigation was stopped. Plant sampling and measurements were regularly carried out as the SWC decreased (see ‘Dehydration tolerance’ section below). The appearance of the plants at field capacity (beginning of the experiment) and after the severe drought treatment (end of the experiment) is illustrated in Supplementary Fig. S4. (iii) ‘Post-drought stage’ (survival): after sampling some tillers of the pots, the remaining plants were immediately rehydrated to field capacity for 10 d to measure the plant survival rate.

### Measurements

#### Dehydration tolerance

As plants of each pot experienced a unique drought intensity (depending on their water consumption based on their leaf area), approximately three different pots of each grass were sampled at 10 successive dates (five different dates for *S. tenacissima* due to the lower number of pots in the experiment) as water deficit increased, namely during the entire plant survival stage (from 100%, i.e. all plants alive, to 0%, i.e. all plants dead, with no visible leaf regrowth). In total, ∼30 pots per grass species were included in the experiment, except for *S. tenacissima*, which had ∼15 pots. Each variable was sampled differently across these pots to capture the variation in plant responses during drought progression. Measurements were carried out from ∼12 tillers per pot that were extracted carefully (cut at the root insertion), immediately taken to the laboratory, and separated into three fractions: (i) the surviving leaf bases (first 20 mm of enclosed leaf bases); (ii) the remaining green aerial tissues; and (iii) the senescent aerial tissues.

The leaf lamina water content (LWC) of fully expanded, green, and mature leaves, and the leaf base water content (LBWC) of the lowest enclosed parts (20 mm) of the leaves, were estimated as follows (Equation 2):


(2) $$\mathrm{LWC}\;(\mathrm{or}\;\mathrm{LBWC})=\left(\frac{\mathrm{FW}-\mathrm{DW}}{\mathrm{FW}}\right)\times 100$$

with FW obtained by weighing leaves (for LWC) and their leaf bases (for LBWC) immediately after sampling, and DW after oven-drying for 48 h at 60 °C (Turner, 1981). Leaf base water potentials (LBWPs, MPa) were measured on leaf bases with psychrometers (ICT International, Armidale, NSW, Australia), which allow measurements down to −10 MPa. Leaf base osmotic potentials (LBOPs, MPa) were measured on leaf bases that have been previously frozen in liquid nitrogen for 15 min, for subsequent determination using psychrometers. LBWP and LBOP could not be measured until the end of the experiment as the most negative values exceeded the limits of the psychrometers (−10 MPa).

Leaf base coefficients of membrane damage (CMD) assess the rate of injury to cell membranes due to drought by measuring the electrolyte leakage from the cells (Blum and Ebercon, 1981). According to Howarth *et al*. (1997), leaf base CMD was calculated as in Equation 3:


(3) $$\mathrm{CMD}=\frac{C1}{C1+C2}\times 100\;$$

where C1 is the conductivity measured with a conductivity probe (CyberScan PC 300 Series Eutech Instruments) after incubation in 18 ml of ultrapure water at 20 °C in the dark for 24 h, and C2 is the conductivity after boiling the samples in 18 ml of ultrapure water for 4 h and cooling for 1 h.

Once sampled, the pots were irrigated until field capacity to allow the remaining plants to rehydrate. Indeed, plant survival cannot be determined before rehydrating the plants since tiller viability cannot be visually determined once most laminae are senescent under drought. After 10 d of rehydration, the number of surviving tillers (i.e. those with at least one regrowing leaf) and the number of dead tillers were counted, to determine the plant survival rate (S) as in Equation 4:


(4) $$\mathrm{Plant}\;\mathrm{survival}\;\mathrm{rate}\;(S)=\frac{\mathrm{Number}\;\mathrm{of}\;\mathrm{surviving}\;\mathrm{tillers}}{\mathrm{Total}\;\mathrm{tiller}\;\mathrm{number}\;}$$

The LD index was calculated following Barkaoui and Volaire (2023). The LD index was derived using response curves of S as a function of SWC (Bolte *et al*., 2016; Barkaoui and Volaire, 2023) according to a logistic function (Equation 5):


(5) $$S=\frac{1}{1+e^{-(\beta_{0}-\beta_{1}\mathrm{SWC})}}$$

where β_0_ and β_1_ described the inflection point and the growth rate of the response curve. LD_50_ is the SWC corresponding to 50% plant mortality. LD_50_ was also expressed as a function of soil water potential (MPa). Similar curve fittings were used to determine the LWC, LBWC, and CMD values associated with 50% plant mortality (LWC_50_, LBWC_50_, and CMD_50_, respectively).

#### Water-soluble carbohydrates

For the identification and quantification of WSCs [mg g^−1^ dry matter (DM)] and the quantification of osmotically active compounds (mosmol g^−1^ DM) in leaf bases, plant samplings were carried out at two dates, the first at field capacity at the beginning of the experiment and the second later under drought (at 3.47% SWC on average). Once collected, the samples were dropped into liquid nitrogen and stored at −80 °C before being freeze-dried at −100 °C for 24 h. Subsequently, they were finely ground, and 50 mg of each was weighed. After three consecutive extractions of WSCs in ultrapure water, the crude extracts were desalted using mini-columns Mobicols (MoBITec, Göttingen, Germany) with ion-exchange resins and polyvinylpolypyrrolidone (PVPP) to remove phenolic compounds (Amiard *et al*., 2003). The purified extract was used to analyse WSCs by HPLC using a refractive index detector (2410 Differential Refractometer, Waters Corporation, Milford, MA, USA). Glucose, fructose, and sucrose were separated on a Sugar-PAK I column (300×6.5 mm, Millipore Waters, Milford, MA, USA) and eluted at 0.5 ml min^−1^ and 85 °C with 0.1 mM Ca-EDTA in ultrapure water. Fructans were separated using a Carbomix Calcium column (300×7.8 mm 8% cross-linkage; Sepax Technologies, Newark, NJ, USA) and eluted with ultrapure water at 0.7 ml min^−1^ and 85 °C. The concentrations of each carbohydrate were determined by comparison with the area of external standards (a mixture of inulin, sucrose, glucose, and fructose at 1 g l^−1^ desalted by passage through a mini-column). To determine the content of osmotically active compounds and the contribution of WSC to osmolarity, the osmolarity of the crude extract and of the purified extract was measured with a freezing point osmometer (Digital Micro-Osmometer, Hermann Roebling Messtechnik, Berlin, Germany).

Distribution of the fructans according to their degree of polymerization (DP) was analysed by high performance anion exchange chromatography and pulsed amperometric detection (HPAEC-PAD ICS-3000, Dionex, CA, USA) equipped with a CarboPac PA1 anion-exchange column (4×250 mm), with elution at 1 ml min^−1^ with 150 mM NaOH and an increasing concentration of sodium acetate. For extracts of *Stipa* species, the increasing concentration of sodium acetate was as follows: 0 mM at 0 min, 50 mM at 6 min, 100 mM at 12 min, 175 mM at 18 min, 250 mM at 19 min, and 500 mM at 30 min. For extracts of *Dactylis* subspecies, the increasing concentration of sodium acetate was as follows: 0 mM at 0 min, 50 mM at 6 min, 100 mM at 12 min, 175 mM at 18 min, 250 mM at 19 min, 425 mM at 30 min, and 500 mM at 60 min.

#### Embolism resistance

Embolism resistance was measured on four leaves for each grass with the optical method (Brodribb *et al*., 2016), which has been demonstrated as a reliable approach for assessing the dynamics of embolism formation in grasses, including reed-like species (West *et al*., 2024). Leaves, attached to their initially well-hydrated plants, were scanned every 5 min for four consecutive days and subjected to increasing water deficit by progressively removing the soil and exposing the roots. The analysis of the image sequence captured during dehydration was performed using ImageJ version 1.43 (National Institutes of Health, Bethesda, MD, USA) to determine the dynamics of loss of relative conductivity. In parallel, plant water potential was measured three times a day using a Schölander pressure chamber (PMS Instrument Company, Corvallis, OR, USA) during the first 3 d of scanning—until −7 MPa. On the fourth day of scanning, water potential was measured on leaf bases using psychrometers reaching −10 MPa (Supplementary Fig. S5). S-shaped curves were fitted between plant water potential and relative conductivity according to a sigmoid function (Pammenter and Van der Willigen, 1998). Embolism resistance was expressed by the water potential values corresponding to 50% or 88% loss of hydraulic conductivity (P_50_ and P_88_). P_50_ and P_88_ are commonly measured thresholds to assess the vulnerability of species to embolism, and they are the metrics most often used to compare hydraulic thresholds between species and populations. Actually, P_88_ was the most discriminant threshold within populations of *D. glomerata* for dehydration tolerance (Volaire *et al*., 2018). In this study on grasses, the hydraulic safety margin could not be measured, while it has been measured in former studies on herbaceous studies, for example by West *et al*. (2024) and Jacob *et al*. (2022), because leaf laminae senesced rapidly after growth cessation, which is reached at ∼4% soil moisture in similar substrates (Barkaoui and Volaire, 2023).

### Statistical analyses

All statistical analyses were performed using R version 4.3.1 (R Core Team, 2023). The plant survival rate was normalized on a scale of 0–1. The response of the plant survival rate to SWC, LWC, LBWC, and CMD was assessed with four generalized linear models (GLMs) with a binomial distribution in which grass identity (species or subspecies) was included as a covariate. A pairwise comparison with a post-hoc Tukey test was performed to identify significant differences in model parameters among grasses using the emmeans and multcomp packages.

Expecting that dehydration of the leaves and leaf bases (meristematic tissues) would dynamically reflect the soil water deficit and trigger plant mortality below a specific threshold for each grass, the specific responses of LWC, LBWC, CMD, and LBWP to SWC were assessed independently with non-linear regressions using the nls2 and nlme packages. A curve based on the $y=a+e^{-b\;\times \mathrm{SWC}}$ function was the best fit for representing LWC and LBWC responses, while $y=a-e^{-b\;\times \mathrm{SWC}}$ was used for CMD and LBWP responses. To optimize curve fitting, data were log-transformed for LBOP as a function of SWC. Similarly, the relationship between CMD and LBWC was analysed, assuming that membrane damage would reflect the dehydration of basal tissues. The effect of species was then assessed by fitting linear models to the predicted values from these non-linear models, and pairwise comparisons were performed using the emmeans package.

The effects of grass identity (species of subspecies) and treatments (irrigation versus drought) on WSCs were tested using two-way ANOVA. When significant, a post-hoc pairwise comparison test (Tukey’s HSD) was conducted using emmeans and multcomp packages to rank the different grasses and treatments.

For embolism resistance, P_50_ and P_88_ were derived from a non-linear model using a bootstrap method with Weibull distribution reparametrized using the fitplc package (Ogle *et al.*, 2009; Duursma and Choat, 2017). Differences in P_50_ and P_88_ between grasses were analysed using two one-way ANOVAs followed, when significant, by a post-hoc pairwise comparison test (Tukey’s HSD) using the emmeans and multcomp packages.

## Results

### Dehydration tolerance

**Fig. 2.**
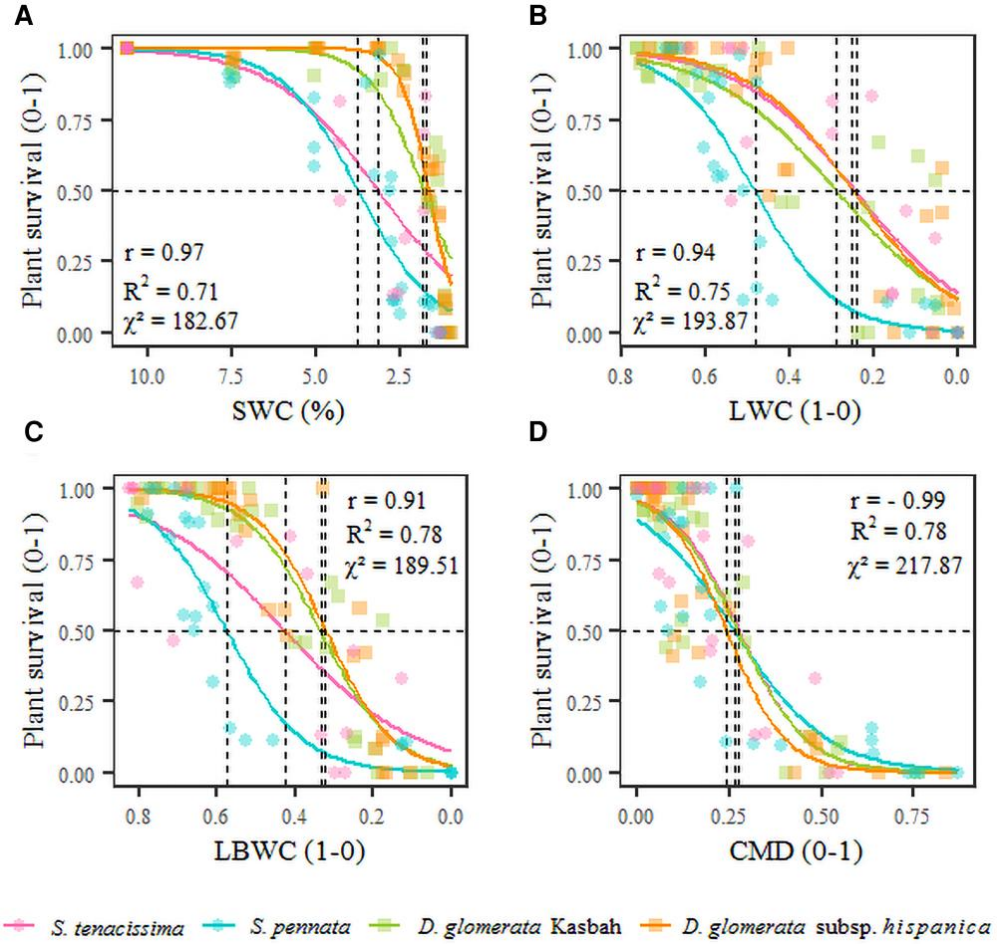
Relationship between variables related to plant survival and plant water status. Plant survival as a function of (A) soil water content (SWC), (B) leaf lamina water content (LWC), (C) leaf base water content (LBWC), and (D) leaf base coefficient of membrane damage (CMD). *n*∼30 per grass, except for *S. tenacissima* (*n*∼15) depending on the variable considered. Symbols: circles for *Stipa* species (pink *S. tenacissima* and blue *S. pennata*); squares for *Dactylis* subspecies (green *D. glomerata* Kasbah and orange *D. glomerata* subsp. *hispanica*). Dashed lines indicate the values that correspond to 50% plant mortality (A–D, respectively). The correlation coefficient (*r*) between plant survival and each variable, the goodness of fit (*R*^2^), and the statistics value (χ^2^) are shown.

Plant survival rate decreased as SWC decreased (*P*<0.001), with differences among grasses. *Stipa pennata* survival showed a steeper decrease in survival than both *Dactylis* subspecies (*P*<0.001), and *S. tenacissima* a steeper decrease than *D. glomerata hisp.* (*P*=0.02) (Fig. 2A). The survival rate also decreased as LWC decreased (*P*<0.001), with a particularly steep decrease for *S. pennata* compared with the other grasses (*P*<0.01) (Fig. 2B). Similarly, the survival rate decreased as LBWC decreased (*P*<0.001), with a steeper decrease for *S. pennata* than for *S. tenacissima* (*P*=0.04) and both *Dactylis* subspecies (*P*<0.001) (Fig. 2C). In contrast, the decrease of plant survival rate (*P*<0.001) with increasing leaf base CMD followed a similar trend for all grasses (Fig. 2D).

The LD_50_, LWC_50_, and LBWC_50_ differed among grasses (*P*<0.001), and were in particular significantly higher for *S. pennata* than for both *Dactylis* subspecies, showing that 50% of *S. pennata* died at a higher SWC (3.8%) with higher tissue water content (57.3%) than both *Dactylis* subspecies, for which the critical thresholds of SWC was ∼1.8% with an LBWC of 33% (Table 1). The two *Stipa* species were more distinct from each other than the two *Dactylis* subspecies. Conversely, CMD_50_ did not differ among grasses (Table 1).

**Table 1.** Lethal drought indices for the perennial grasses, *S. tenacissima*, *S. pennata*, *D. glomerata* Kasbah, and *D. glomerata hisp*.

Species/subspecies	LD_50_ (%)	LWC_50_ (%)	LBWC_50_ (%)	CMD_50_ (%)
*S. tenacissima*	3.18 ab (= −3.07 MPa)	24.10 a	42.70 a	27.50 a
*S. pennata*	3.79 a (= −2.28 MPa)	48.20 b	57.30 b	26.40 a
*D. glomerata* Kasbah	1.84 bc (= −5.90 MPa)	28.90 a	33.40 a	27.50 a
*D. glomerata hisp.*	1.72 c (= −6.25 MPa)	25.10 a	32.30 a	24.20 a

Indices were calculated using soil water content (LD_50_) expressed in % and MPa, leaf lamina water content (LWC_50_), leaf base water content (LBWC_50_), and leaf base coefficient of membrane damage (CMD_50_) expressed in % corresponding to 50% tiller survival. Lower case letters indicate significant differences between grasses.

**Fig. 3.**
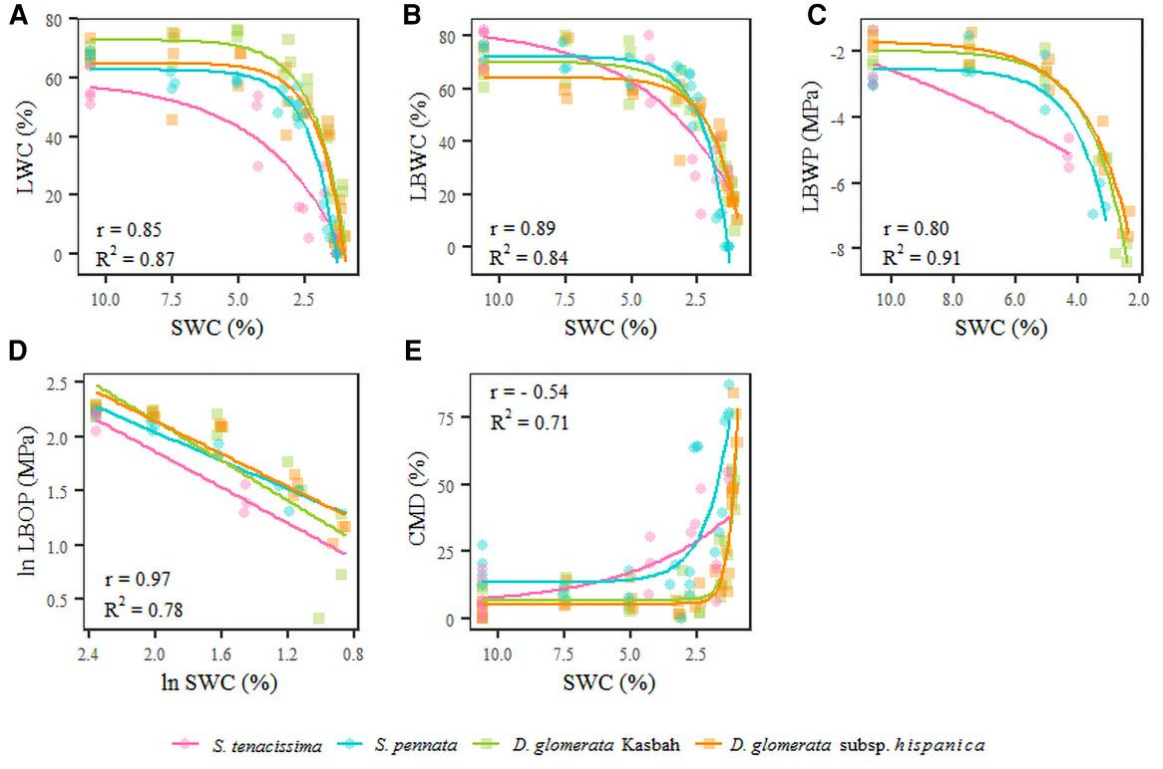
Relationship between variables related to plant water status and soil water content. (A) Leaf lamina water content (LWC), (B) leaf base water content (LBWC), (C) leaf base water potential (LBWP), (D) log-transformed leaf base osmotic potential (ln LBOP), and (E) leaf base coefficient of membrane damage (CMD) as a function of soil water content (SWC). *n*∼30 per grass, except for *S. tenacissima* (*n*∼15), depending on the variable considered. Symbols: circles for *Stipa* species (pink *S. tenacissima* and blue *S. pennata*); squares for *Dactylis* subspecies (green *D. glomerata* Kasbah and orange *D. glomerata* subsp. *hispanica*). The correlation coefficient (*r*) between each variable and SWC, and the goodness of fit (*R*^2^) are shown.

LWC, LBWC, LBWP, and LBOP all decreased with decreasing SWC (*P*<0.001), although there were no differences between grasses (Fig. 3A–D). The most negative measured LBOPs were −7.37 MPa for *S. tenacissima*, −7.28 MPa for *S. pennata*, −9.62 MPa for *D. glomerata* Kasbah, and −8.26 MPa for *D. glomerata hisp.* (Fig. 3D). In contrast, leaf base CMD increased as SWC decreased (*P*<0.001), with *S. tenacissima* being significantly different from the two subspecies of *Dactylis* (*P*<0.01) (Fig. 3E). In addition, leaf base CMD increased as LBWC decreased (*P*<0.001), although there were no differences between grasses (Supplementary Fig. S6).

### Water-soluble carbohydrates in leaf bases

**Fig. 4.**
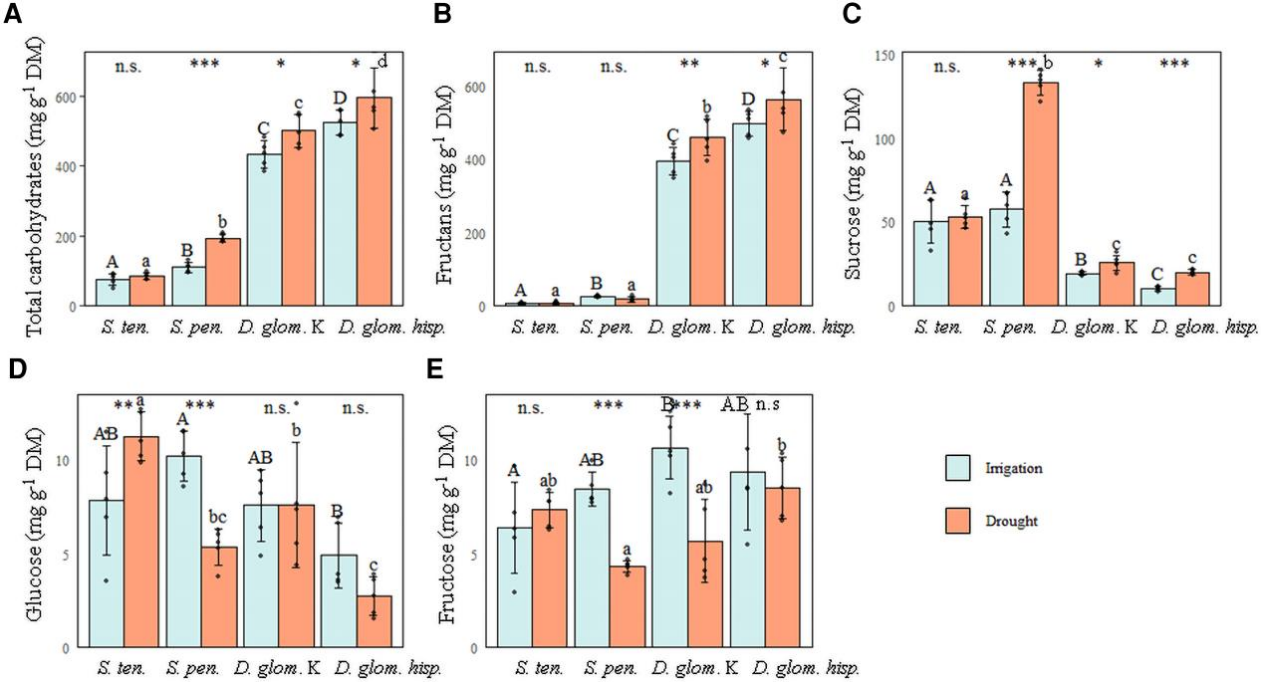
Concentration of soluble carbohydrates in leaf bases. Concentration (mg g^−1^ DM) of (A) total soluble carbohydrates, (B) fructans, (C) sucrose, (D) glucose, and (E) fructose for the four studied perennial grasses, *S. tenacissima*, *S. pennata*, *D. glomerata* Kasbah, and *D. glomerata hisp*. Blue bars (irrigation, field capacity, SWC=10.6%, *n*=5 per grass) and orange bars (drought, SWC=3.47%, *n*=5 per grass). Error bars represent ±SD. Upper case letters indicate the comparison between grasses under irrigation, and lower case letters under drought, using Tukey’s test. Asterisks or n.s. indicate the differences between irrigation and drought within each grass (***P≤0.001; **P≤0.01; *P≤0.05; n.s.=non-significant, *P*>0.05).

Total WSC concentration was much higher in *Dactylis* subspecies than in *Stipa* species (Fig. 4A). Fructans were the predominant WSC in both *Dactylis* subspecies, while it was sucrose in both *Stipa* species (Fig. 4B, C). *Dactylis glomerata hisp.* accumulated significantly more fructans than *D. glomerata* Kasbah (531 mg g^−1^ DM versus 428 mg g^−1^ DM *P*<0.05, Fig. 4B) while this was the opposite for sucrose contents (15 mg g^−1^ DM versus 22 mg g^−1^ DM, *P*<0.05, Fig. 4C). *Stipa* species were also able to synthesize fructans (Supplementary Fig. S7), but to a very low level, namely <7 mg g^−1^ DM in *S. tenacissima* and 21 mg g^−1^ DM in *S. pennata* (Fig. 4B). Both *Dactylis* subspecies synthesized fructans with a high DP, up to 90 for *D. glomerata hisp.* and >DP 90 for *D. glomarata* Kasbah (Supplementary Fig. S7B), while the higher DP was ∼20 for the two *Stipa* species, with very low levels for *S. tenacissima* (Supplementary Fig. S7A). Total WSC concentration was higher under drought than under the irrigation regime, except for *S. tenacissima* for which the WSC concentration remained very low (Fig. 4A). In *Dactylis* subspecies, this effect was mainly due to an increase in fructan content (461 mg g^−1^ DM for *D. glomerata* Kasbah and 564 mg g^−1^ DM for *D. glomerata hisp.*, Fig. 4B) and to a lesser extent to an increase in sucrose content (26 mg g^−1^ DM and 20 mg g^−1^ DM, respectively, Fig. 4C). In *Stipa* species, this effect was due to a high accumulation of sucrose (133 mg g^−1^ DM for *S. pennata* and 53 mg g^−1^ DM for *S. tenacissima*, Fig. 4C). Glucose and fructose contents were <15 mg g^−1^ DM in all grasses, whatever the conditions (Fig. 4D, E). Glucose content was not significantly affected by drought in *Dactylis* subspecies. In *S. tenacissima*, glucose content increased under drought, while it decreased in *S. pennata* (Fig. 4D). Fructose content was not affected by drought in *S. tenacissima* and *D. glomerata hisp*., while it decreased significantly under drought in *D. glomerata* Kasbah and *S. pennata* (Fig. 4E).

**Fig. 5.**
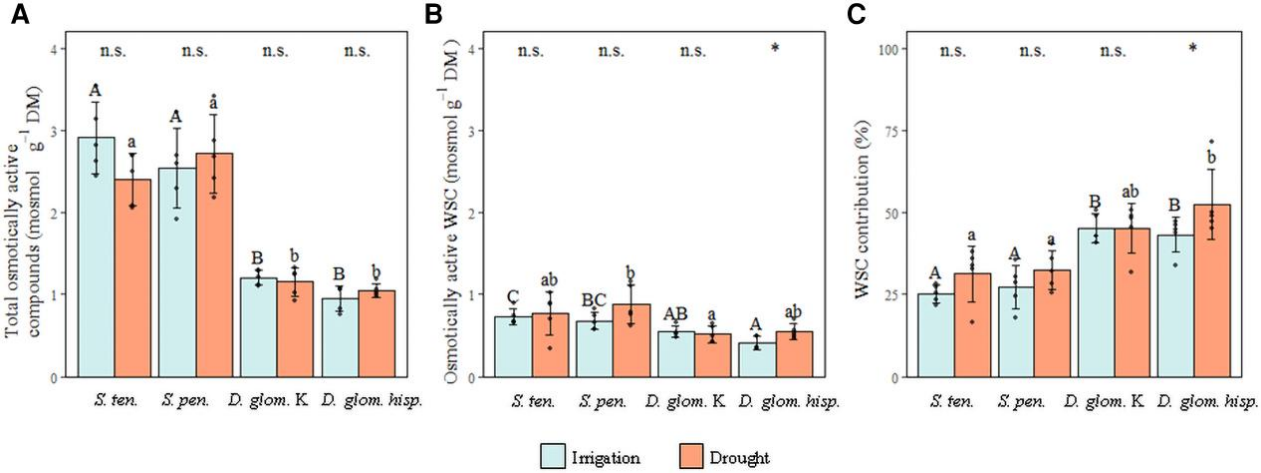
Osmotic composition of leaf bases. (A) Total osmotically active compounds (mosmol g^−1^ DM), (B) osmotically active WSC (mosmol g^−1^ DM), and (C) contribution of WSC to total osmotically active compounds in the leaf bases (%) for the four studied perennial grasses, *S. tenacissima*, *S. pennata*, *D. glomerata* Kasbah, and *D. glomerata hisp*. Blue bars (irrigation, field capacity, SWC=10.6%, *n*=5 per grass) and orange bars (drought, SWC=3.47%, *n*=5 per grass). Error bars represent ±SD. Upper case letters indicate the comparison between grasses under irrigation, and lower case letters under drought using Tukey’s test. Asterisks or n.s. indicate the differences between irrigation and drought within each grass (***P≤0.001; **P≤0.01; *P≤0.05; n.s.=non-significant, *P*>0.05).

The total content of osmotically active compounds in leaf bases was much higher in *Stipa* species than in *Dactylis* subspecies (2.65 mosmol g^−1^ DM versus 1.09 mosmol g^−1^ DM, respectively; *P*<0.001) (Fig. 5A). This was also observed for the content of osmotically active WSC, but the difference between the two genera was far less pronounced (0.764 mosmol g^−1^ DM versus 0.505 mosmol g^−1^ DM, respectively; *P*<0.001) (Fig. 5B). Consequently, the contribution of WSC to the osmotically active compounds was higher in *Dactylis* subspecies than in *Stipa* species (46.6% versus 29.1%, respectively; *P*<0.001) (Fig. 5C). The total content of osmotically active compounds was not affected by drought, either in *Stipa* species or in *Dactylis* subspecies (Fig. 5A). The same was observed for the content of osmotically active WSCs and their contribution to the total of osmotically active compounds, with the exception of *D. glomerata hisp.*, for which they were slightly but significantly higher under drought (Fig. 5B, C).

### Embolism resistance

For embolism resistance measurements, four leaves per grass were initially targeted; however, embolism was expressed in only three leaves for *S. tenacissima* and *D. glomerata hisp*. The leaf P_50_ of *S. tenacissima* was the most negative (−9.36 MPa) and significantly more negative than the P_50_ of *S. pennata* (−8.10 MPa). Both species exhibited more negative values than those of the *Dactylis* subspecies, with a P_50_ value around −2.9 MPa (*P*<0.001). The same trends were found for P_88_ (Table 2). Additionally, the raw vulnerability curves are shown in Supplementary Fig. S8.

**Table 2.** Mean values of P_50_ and P_88_ and their 95% confidence intervals (CIs) for the perennial grasses, *S. tenacissima* (*n*=3), *S. pennata* (*n*=4), *D. glomerata* Kasbah (*n*=4), and *D. glomerata hisp*. (*n*=3)

	Mean	CI		Mean	CI	
Species/subspecies	P_50_ (MPa)	P_50_ (2.5%)	P_50_ (97.5%)	P_88_ (MPa)	P_88_ (2.5%)	P_88_ (97.5%)
*S. tenacissima*	−9.36 a	−9.35	−9.37	−9.42 a	−9.41	−9.43
*S. pennata*	−8.10 b	−8.08	−8.12	−8.44 b	−8.40	−8.47
*D. glomerata* Kasbah	−2.89 c	−2.87	−2.90	−3.09 c	−3.06	−3.12
*D. glomerata hisp.*	−2.92 c	−2.91	−2.93	−3.13 c	−3.12	−3.15

Lower case letters indicate significant differences between grasses.

## Discussion

This study investigated the main strategies of perennial grasses from Mediterranean and semi-arid rangelands to survive severe drought. We analysed both their capacity to tolerate dehydration in relation to WSC accumulation and embolism resistance, two strategies that proved to be uncorrelated when comparing four perennial grasses of *Stipa* species and *Dactylis* subspecies.

### Dehydration tolerance and water-soluble carbohydrates in leaf bases

The plant tolerance to soil water deficit was high overall among the four studied grasses, as plants reached 50% mortality (LD_50_) when the soil water potential was around −2.68 MPa (3.49% SWC) for the *Stipa* species, and even more negative, at −6.08 MPa (1.78% SWC), for the *Dactylis* subspecies (Table 1). These results highlight that the ‘permanent wilting point’ (−1.5 MPa), classically used as a benchmark for plant water stress (Tolk, 2003), greatly underestimates the drought survival capacity of perennial grasses. Unlike annual species, which usually cease growing and die near this standard threshold, the studied perennial grasses can withstand much more negative soil water potential before reaching lethal dehydration levels (Volaire, 2003). The tolerance to soil water deficit of both *Dactylis* subspecies was even higher than what was previously found for a Mediterranean cultivar of *Dactylis* (Medly) for which LD_50_ equalled −4.47 MPa (2.19% SWC) under comparable environmental conditions (Barkaoui and Volaire, 2023). This study reports, for the first time, an extreme level of tolerance to soil water deficit achieved by the Moroccan subspecies (−5.90 MPa, 1.84% SWC) and the Spanish subspecies of *D. glomerata* (−6.25 MPa, 1.72% SWC).

From the LD_50_ only, the correlation between the aridity level of the site of origin and dehydration tolerance is inconclusive and would require comparison of a wider range of species and subspecies. However, the highest values of leaf base membrane damage (CMD) (Fig. 3E) and mortality thresholds found for *S. pennata*, the species from the least arid environment, suggest that it is the most vulnerable and dies at a higher LWC_50_ (i.e. is less dehydration tolerant) than the other grasses from more arid environments (Fig. 2B).

*Dactylis* subspecies were both more tissue dehydration tolerant than the two *Stipa* species, with a 50% survival rate reached at an LBWC of 32.8% compared with 50.0% for the *Stipa* species (Table 1). Although all grasses reached very low recordable osmotic potential (i.e. below −7.3 MPa in their leaf bases), extreme values were recorded in these surviving organs of *Dactylis* subspecies (down to −9.6 MPa) (Fig. 3D). These levels are far below osmotic potentials measured in leaves of other grasses under drought, for instance −3.2 MPa in the arid grass *Leptochloa fusca* (Ashraf and Yasmin, 1995) or −3 MPa in the grass *Bouteloua gracilis* (Bushey *et al*., 2023). Our results suggest that a low osmotic potential in surviving meristematic tissues—the leaf bases—contributes to maintaining higher hydration by limiting water loss out of the cells, by diverting the whole plant water to the leaf bases, and/or by improving water extraction from the soil even under severe water deficit (Ramírez *et al*., 2007). This may enhance drought survival of very small organs with limited water flow, in particular in the *Dactylis* subspecies. In contrast to mature lamina, meristems represented a strong sink for water and carbohydrates throughout severe drought (Spollen and Nelson, 1994), as also recently demonstrated in basal axillary buds in pea (Ray *et al*., 2025). Our results confirm the importance of measuring the suitable organs (leaf bases rather than lamina) to understand dehydration tolerance in grasses (Barkaoui and Volaire, 2023).

The grasses accumulated different levels of WSCs in their leaf bases, reflecting that WSC accumulation and composition are species/subspecies-specific traits influenced by drought conditions (Fig. 4). Fructans, detected in high concentrations in both *Dactylis* subspecies, with even higher levels under drought, may act as osmoprotectants, helping to maintain cell turgor (Livingston *et al*., 2009). However, despite this very high amount of fructans, total WSC contributed to <50% of the osmotically active compounds (Fig. 5C). This indicates that the fructans accumulated in *Dactylis* subspecies are highly polymerized molecules that contribute relatively little to osmolarity and thus to osmoprotection. Due to their ability to insert between the head groups of membrane lipids (Vereyken *et al*., 2001) and to act as antioxidants (Stoyanova *et al*., 2011), they may also contribute to membrane stability (Hincha *et al*., 2007), thus ensuring the survival of the plant bases during periods of drought through dehydration tolerance (Volaire *et al*., 2014, 2020; Zwicke *et al*., 2015). Furthermore, accumulated fructans may serve as an energy reserve, maintaining metabolic functions and cell survival when photosynthesis is impaired due to reduced water availability and limited stomatal opening (White, 1973). Indeed, the fact that the *Dactylis* subspecies sheds its leaves may lead not only to reduced water loss but also to the relocation of sugars to the leaf bases, thereby protecting meristematic cells. Although most of the leaves were senescent, plants of these subspecies can stay alive under intense drought (Volaire *et al*., 2009, 2018, 2020).

### Embolism resistance

Embolism resistance of plants differed strongly between *Stipa* species and *Dactyli*s subspecies. The P_50_ of *Stipa* species was twice as low as that of the *Dactylis* subspecies, revealing their particularly high embolism resistance (P_50_= −9.36 MPa for *S. tenacissima* and P_50_= −8.10 MPa for *S. pennata*) (Table 2). These embolism thresholds exceed those reported in other grasses, such as −4.4 MPa to −6.1 MPa in five pasture grasses measured with the optical vulnerability method (Jacob *et al*., 2022), −0.5 MPa to −7.5 MPa in perennial grasses using the centrifuge technique (Lens *et al*., 2016), and −0.57 MPa to −4.78 MPa in graminoids (*Poaceae*, *Cyperaceae*, and *Juncaceae*) and forbs measured with various techniques (Huang *et al*., 2024). This result suggests that *Stipa* species have specialized in embolism resistance as a primary strategy to survive severe drought.

In this study, *S. tenacissima* originating from a more arid site showed significantly more negative P_50_ values than *S. pennata*. Our results reveal for the first time that the embolism resistance of a perennial grass from semi-arid areas can exceed −9 MPa, underlining the ability of both *Stipa* species to maintain hydraulic functions at extreme negative pressures. Previous studies showed that *S. tenacissima* has a highly efficient capacity for resource uptake and use (Soliveres *et al*., 2010, 2013); moreover, the partially buried basal organs in the soil and the high lignification of its leaf tissues contribute to protecting the leaf bases of *S. tenacissima* plants (Belkhir *et al*., 2012) and to limiting water loss (Bochet *et al*., 2000). In addition, high lignification and leaf narrowness could result in low vein density per unit leaf area, which may positively contribute to water transport efficiency, as observed in woody species (Brodribb *et al*., 2007; Sack and Scoffoni, 2013).

The viability of root apices contributed to drought survival in temperate grass species (Zwicke *et al*., 2015). Hence, in field conditions, S*tipa* species may survive more by maintaining the viability of root apices than that of leaf meristems. Embolism resistance should therefore be tested in the roots of *Stipa* species. Our study, carried out in small pots with restricted rooting depth, was not suitable to test this hypothesis. In woody species, the physiological vulnerability to drought-induced mortality was shown to result from three possible failure modes, namely carbon starvation, hydraulic failure, and phloem transport failure (Mencuccini *et al*., 2015), suggesting that a diversity of mechanisms may also contribute to drought mortality in grasses. However, our results confirm that the mechanisms of drought survival differ between woody and herbaceous species. Indeed, the positive relationship between the high turgor loss point and drought survival in herbaceous grassland species was found to be opposite to the negative relationship previously established in woody plants (Sun *et al*., 2020).

Regarding *Dactylis* subspecies, our results differed from those reported by Volaire *et al*. (2018) for *D. glomerata* Kasbah from Morocco, which was twice as negative in this previous study (P_50_ of −6.36 MPa versus −2.89 MPa). These differences found for the same subspecies could be due to differences in the measurement technique employed, the part of the plant, and/or the phenological phase. We used an optical technique on leaves of vegetative tillers, in contrast to Volaire *et al*. (2018) who used a cavitation technique that consisted of subjecting floral stems to centrifugal force to impose increasing pressures and measure embolism in lignified stems. In our case, plants were vegetative and analysed in spring, while in the former study, plants were at the reproductive stage in early summer, with lignified stems that may have conferred a higher embolism resistance when measured in those tissues. Previous work on tree species showed no differences in embolism resistance between the two measurement techniques (Lamarque *et al*., 2018). However, we show that embolism resistance in herbaceous plants may depend on the type of measurement, the studied organ, plant phenology, and the functional continuum between leaf blades and leaf bases. In species that shed their leaves during drought, such as *Dactylis* subspecies, embolism resistance may contribute less to survival than in species with tough leaves that remain attached throughout the drought, as is the case for *Stipa* species. This should be experimentally investigated, particularly under field conditions.

### Surviving drought requires a combination of strategies

This study on perennial grasses from semi-arid areas, conducted under controlled water availability conditions, revealed a species/subspecies-specific combination of drought survival strategies to adapt to the environmental conditions of their origin. In *Dactylis* subspecies, the concentration of WSCs was high, contributing to almost 50% of the osmotically active compounds, which helped to maintain cell turgor and function at low water potentials, thereby compensating to some extent for their lower leaf embolism resistance. Conversely, *Stipa* species appeared to rely more on maintaining hydraulic integrity through highly embolism-resistant xylem, which may be ascribed to a probably greater carbon allocation to tissue lignification.

However, despite their high embolism resistance and high content of osmotically active compounds, *Stipa* species reached lethal leaf base dehydration at a relatively higher SWC, reflecting the lower tolerance of these tissues to dehydration. This suggests that the low contribution of WSCs to the osmotically active compounds limits the cell protection under high tissue dehydration. Moreover, this is consistent with the findings of West *et al*. (2024), who emphasized that persistence in dry environments cannot be explained solely by the evolution of highly embolism-resistant xylem, but also requires additional strategies such as dehydration avoidance to understand plant adaptation. This combination of strategies illustrates how grasses can adopt alternative mechanisms to cope with drought stress, and shows that no single strategy fully determines survival under soil water deficit.

Since drought survival combines multiple complementary strategies, field studies accounting for seasonal photosynthetic activity and growth patterns in winter and spring, phenological traits (flowering date for dehydration escape and summer dormancy), as well as rooting depth (dehydration avoidance) and the viability of root apices will be required to identify the maximum aridity thresholds that could jeopardize the survival of even the most adapted perennial herbaceous species.

In addition, we consider it essential to conduct further research using standardized methodologies across a wider range of species and populations to enhance the comparability of results and improve our understanding of drought survival strategies and hydraulic traits. Such knowledge will be key to predicting the future adaptation and persistence of Mediterranean grasslands.

## Supplementary Material

erag003_Supplementary_Data

## Data Availability

The data that support the findings of this study are openly available in figshare at https://doi.org/10.6084/m9.figshare.30917117.

## Supplementary data

The following supplementary data are available at *JXB* online.

Table S1. Geographical data of the collection sites for the four studied perennial grasses.

Fig. S1. Geographical location of the collection sites of the four studied perennial grasses.

Fig. S2. Environmental variables in the glasshouse during the experiment.

Fig. S3. Variation in soil water content over time in pots.

Fig. S4. Photographs of plants at soil field capacity and at the end of the severe drought period.

Fig. S5. Experimental design for the measurement of embolism resistance.

Fig. S6. Leaf base coefficient of membrane damage as a function of leaf base water content.

Fig. S7. Distribution of the fructans according to their degree of polymerization.

Fig. S8. Raw vulnerability curves for P_50_ and P_88_.
