# Supplementary material for: Semi-arid grasses combine contrasting strategies of dehydration tolerance associated with carbohydrate storage and embolism resistance under drought
Source: J Exp Bot. 2026 Feb 24;77(8):2443–55. doi: 10.1093/jxb/erag003 (PMC13080357; doi:10.1093/jxb/erag003)
Supplement: erag003_Supplementary_Data [file erag003_supplementary_data.docx]

**SUPPORTING INFORMATION**

**Table S1.** Geographical data of the collection sites for the four studied perennial grasses. Climatic data from 2002 to 2022 for *S. tenacissima*, *S. pennata* and *D. glomerata* subsp. *hispanica*. Climatic data from 1954, year of *D. glomerata* Kasbah collection.

| Site | *S. tenacissima* | *S. pennata* | *D. glomerata* Kasbah | *D. glomerata* *hisp.* |
| --- | --- | --- | --- | --- |
| Location | Alicante, Spain | Larzac, France | Oum-Er-Rbia Valley, Morocco | Seville, Spain |
| Latitude | 38º 20' 42.61'' N | [43º 55' 45.09'' N](https://fr.wikipedia.org/wiki/Causse_du_Larzac#/maplink/1) | 32° 43' 47.00'' N | 37° 22' 58.19'' N |
| Longitude | 0º 28' 53.36'' W | 3º 06' 22.26'' E | 7° 55' 52.00'' W | 5° 58' 23.41'' W |
| Altitude (m) | 3 | 800 | 252 | 35 |
| Mean annual temperature (°C) | 18.9^a^ | 10.1^b^ | 11.7^c^ | 19.8^a^ |
| Total annual precipitation (mm) | **256.9^a^** | 944.8^b^ | 270^c^ | 519.8^a^ |
| Aridity index^d^ | **8.89** | 47.00 | 12.44 | 17.44 |

^a^Meteorological stations of Alicante (08359) and Sevilla San Pablo (08391). Data from https://www.infoclimat.fr/

^b^Meteorological station La Fage, INRAE.

^c^Oram (1990).

^d^$AI=\frac{Precipitation}{Temperature+10 ºC}$ according to De Martonne (1926).

**Figure S1.** Geographical location of the collection sites of the four studied perennial grasses, *S. tenacissima* L. from Alicante (South-Eastern Spain), *S. pennata* L. from Larzac (Southern France), *D.* *glomerata* L. (Kasbah) from Oum-Er-Rbia (Western Morocco) and *D. glomerata* L. subsp. *hispanica* (Roth) Nyman from Seville (Southern Spain).

**
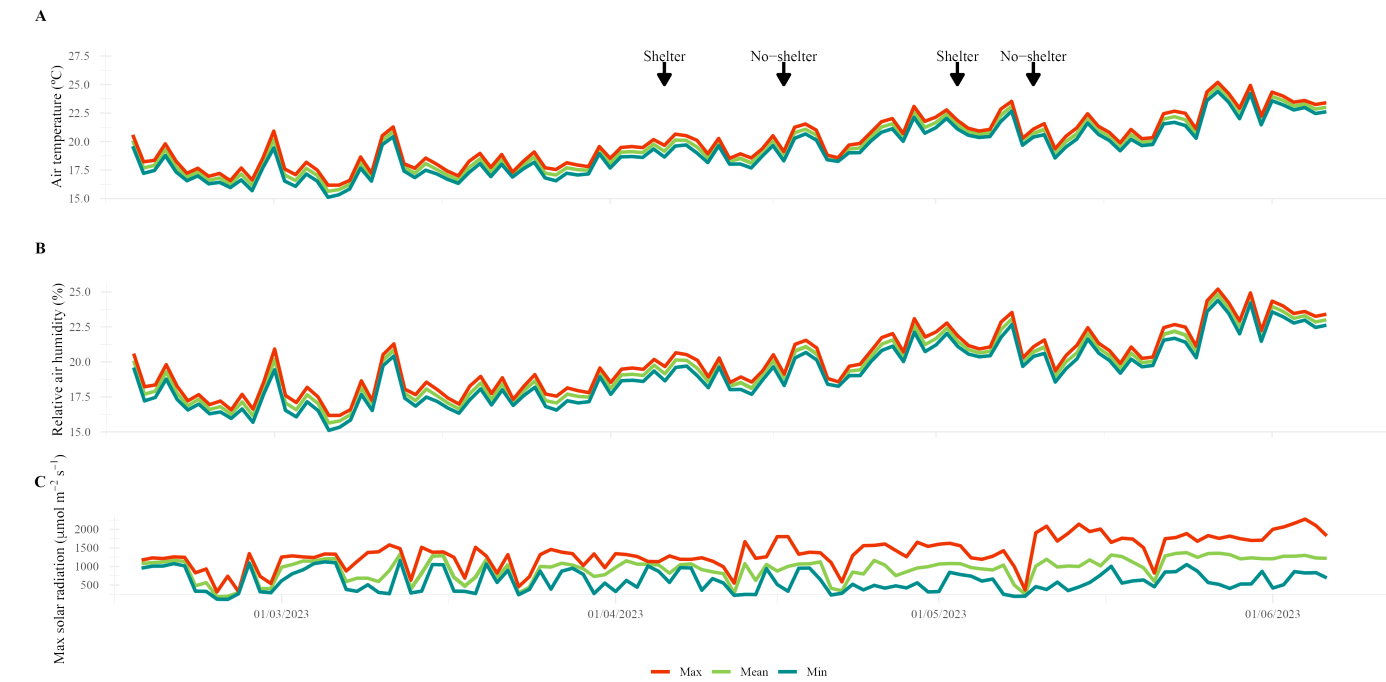
**

**Figure S2.** Environmental variables in the glasshouse during the experiment. Daily climatic variables from mid-February to early June 2023. (A) Air temperature (ºC), (B) relative air humidity (%), (C) and maximum solar radiation (µmol m^-2^ s^-1^) during the sunshine period. Red, green and blue lines indicate the maximum, mean and minimum daily values respectively. Arrows show the periods when the top of the glasshouse was covered by a light shelter in order to reduce the inside temperature.


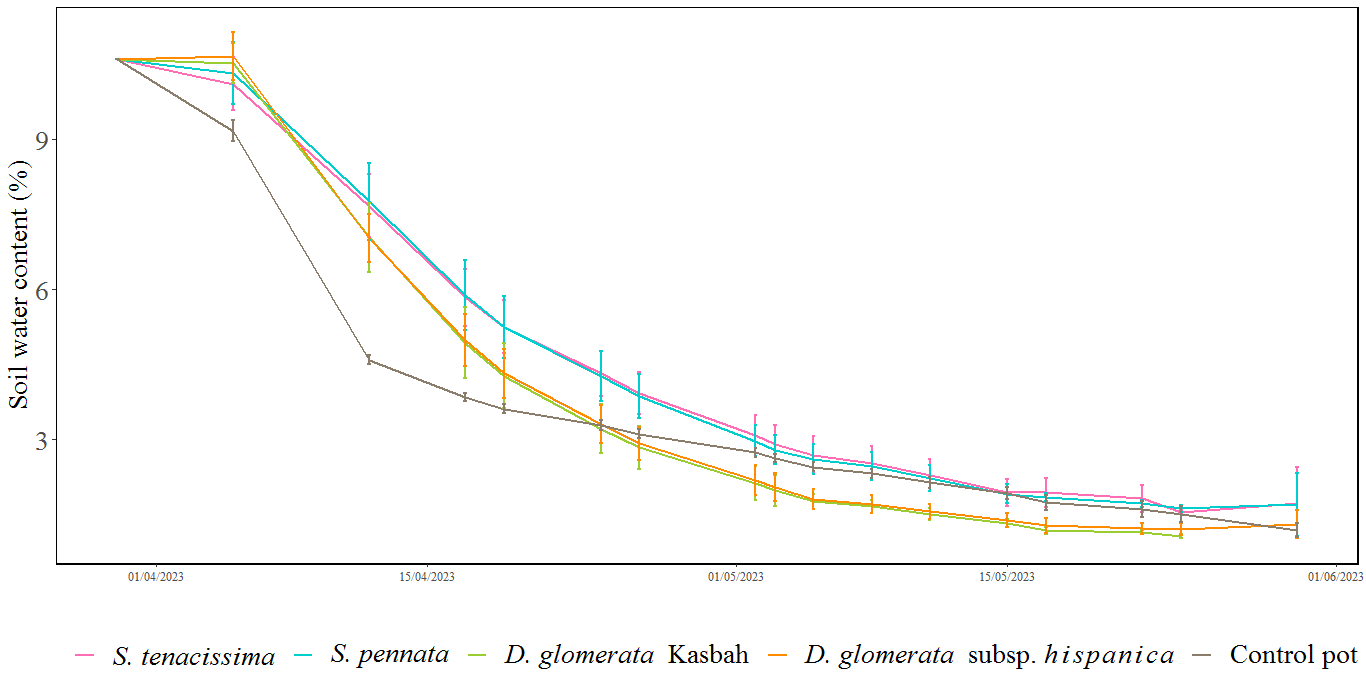


**Figure S3.** Variation in soil water content over time in pots n~30 per grass, except for S. tenacissima (n=15). Soil water content starts at 10.6% (field capacity) and ends at 1% (death of all plant material). The loss of soil water content in pots containing *S. tenacissima* (pink), *S. pennata* (blue), *D. glomerata* Kasbah (green), *D. glomerata* subsp. *hispanica* (orange) and control pot (grey).


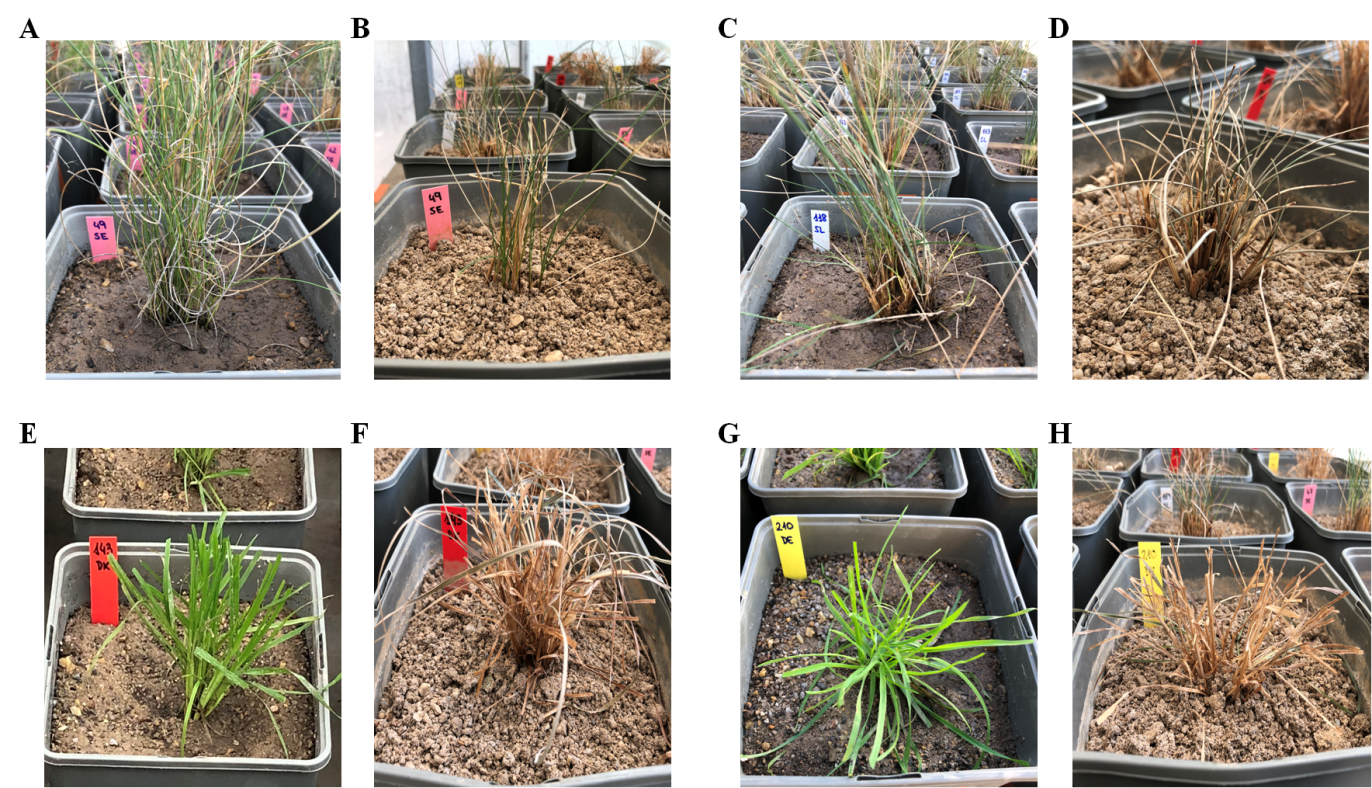


**Figure S4.** Photographs of four studied perennial grasses under two water availability conditions: at field capacity (left) and at the end of the severe drought period (right). (A–B) *S. tenacissima*, (C–D) *S. pennata*, (E–F) *D. glomerata* Kasbah, (G–H) *D. glomerata* subsp. *hispanica*.

**Figure S5.** Experimental design for the measurement of embolism resistance in four Mediterranean perennial grasses, *S. tenacissima*, *S. pennata*, *D.* *glomerata* Kasbah and *D. glomerata* subsp. *hispanica*: (1) Irrigation at field capacity; (2) Detection of embolised areas by scanning a leaf attached to the plant outside the pot every 5 minutes for 7 days (n=4 per grass); (3) Measurement of water potential in leaves during the first 3 days by Schölander pump (up to -7 MPa) and in leaf bases during the following 4 days by psychrometers (up to -10 MPa); (4) Correlation between relative conductivity and xylem pressure values.

**
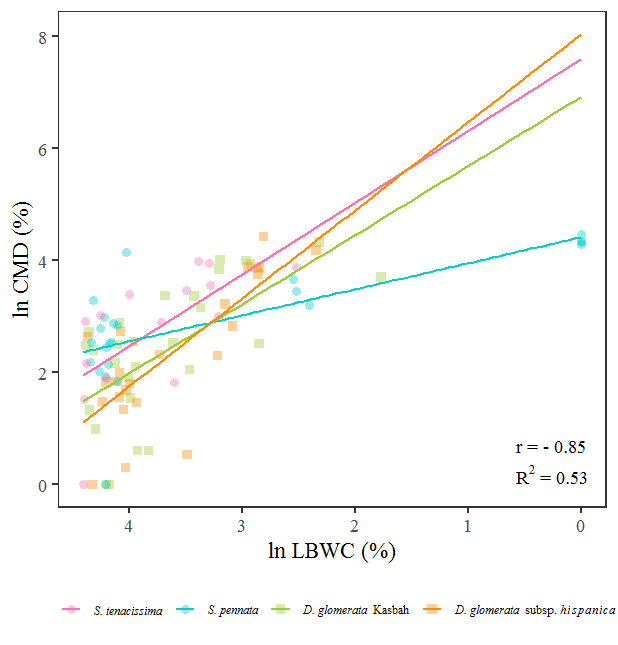
**

**Figure S6.** log-transformed leaf base coefficient of membrane damage (ln CMD) as a function of log-transformed leaf base water content (ln LBWC). n~30 per grass (depending on the species/subspecies), except for S. tenacissima (n=15). Symbols: circles for *Stipa* species (pink *S. tenacissima* and blue *S. pennata*); squares for *Dactylis* subspecies (green *D. glomerata* Kasbah and orange *D. glomerata* subsp. *hispanica*). The correlation coefficient (r) between leaf base coefficient of membrane damage and leaf base water content, and the goodness of fit (R^2^) are shown.

**Figure S7.** Distribution of the fructans according to their degree of polymerization (DP). HPAEC-PAD chromatograms of water soluble carbohydrates in leaf meristems of (A) *Stipa* species and (B) *Dactylis* subspecies sampled from plants under irrigation. Range of DP are indicated below the chromatograms. Chromatogram of a standard of inulins from chicory (*Cichorium intybus*) obtained with the same HPAEC-PAD conditions is given for comparison.

**Figure S8.** Raw vulnerability curves for P_50_ (panels A–D) and P_88_ (panels E–H), along with their 95% confidence intervals (2.5%–97.5%), for the four studied perennial grasses, *S. tenacissima* (n=3), *S. pennata* (n=4), *D. glomerata* Kasbah (n=4), and *D. glomerata* subsp*. hispanica* (n=3).
